# Open data for evolutionary synthesis: an introduction to the NESCent collection

**DOI:** 10.1038/sdata.2014.30

**Published:** 2014-09-02

**Authors:** Todd J. Vision, Karen Cranston

**Affiliations:** 1 National Evolutionary Synthesis Center, Durham, North Carolina, USA; 2 Department of Biology, University of North Carolina, Chapel Hill, North Carolina, USA; 3 Department of Biology, Duke University, Durham, North Carolina, USA

Many of the historic turning points in the history of evolutionary science are examples of ‘synthetic research’, in which new knowledge was generated through the integration of existing data, methods, results and concepts^1^. This tradition goes back to Darwin’s famously multifaceted case for evolution by natural selection in *The Origin of Species*, the reconciliation of Mendelian and statistical genetics by R.A. Fisher^2^, and the Modern Evolutionary Synthesis of the mid-20th century that brought together population genetics, paleontology and other schools of evolutionary theory^3^.

For the past decade, with funding from the US National Science Foundation (NSF), National Evolutionary Synthesis Center (NESCent) has promoted the continuation of this tradition through competitive support for researchers who wish to apply synthesis to any area of evolutionary science of their choosing. While the center runs several programs, the ones directly responsible for the most research outputs are the resident scholars (graduate, postdoctoral and sabbatical fellows) and working groups (with recurrent face to face meetings over two years with participants from a diversity of disciplines and from institutions around the world). These programs provide researchers with opportunities to pursue ideas for synthetic research that are much more difficult to fund through traditional channels^4^.

Data are both a critical input and output for many synthetic investigations. Projects supported by the center often invest a great deal of effort in the collection, curation, and integration of existing data for meta-analysis, even though, as a matter of policy, NESCent does not support the collection of new data. In other cases, worthwhile research projects have had to be declined at the proposal stage to due to the inaccessibility of previously collected data for reuse.

In order to make it possible for future researchers to build upon the work being done today, NESCent has had a policy since 2006 that data created through NESCent-sponsored activities be open (http://nescent.org/public_documents/Informatics_Policy/Data_and_Software_Policy.pdf). More precisely, the policy stipulates that all data (and software) are to be made publicly available no later than one year after the conclusion of the NESCent award or immediately upon publication of the results, whichever comes earlier. Data should be deposited in a public data repository with no restrictions on use and dissemination beyond the form of attribution. Furthermore, the data should be adequately documented for validation and reuse, including appropriate attribution of its original source. To assist in seeing this policy carried through, NESCent has provided support ranging from consultation (e.g., metadata standards or licensing) to digitization to the provision of specialized tools for collaborative data wrangling.

Recognizing that a policy that applies only to NESCent supported research would have a relatively limited impact, NESCent contributed to adoption of the Joint Data Archiving Policy (JDAP) by many key journals in evolution and ecology beginning in 2011 (http://datadryad.org/pages/jdap). An important role played by the center was incubating the Dryad Digital Repository, which now provides a trusted home for data associated with the scholarly record for which a specialized long-term repository is lacking^5^, including much of the data described in this Collection. The JDAP and the advent of Dryad have been responsible for a dramatic uptick in the availability of data associated with traditional publications in the field^6^.

However, a traditional publication is not the best vehicle for dissemination when the data themselves make a standalone contribution to scholarship. This rolling Collection provides an outlet for NESCent-sponsored researchers to publish uniquely valuable data in a coherent, independently citable package, carefully described with standardized metadata. All the Data Descriptors are linked to preservation snapshots of the data in a form that will be reusable for years to come and, in some cases, these are complemented by more detailed or more dynamic data access mechanisms. The first four Data Descriptors to be published in the Collection nicely illustrate some of the diversity of data outputs generated through NESCent-sponsored activities, including literature-based data compilations, the results from long-term experimental and observational programs, and digitized historic records.

One common mode of synthetic research is to compile data points that have been reported at various places in the literature into a single dataset that is amenable to comparative analysis or meta-analysis, as exemplified by the contribution from the Tree of Sex Consortium^7^. The 17 authors in the Consortium, participants in a NESCent Working Group, wished to understand the drivers behind the diversity of genetic and environmental sex determination systems in nature. Collectively, they had expertise in several different taxonomic groups and a number of different aspects of sex determination. This range of expertise allowed them to compile a high-quality dataset of more than 20 variables about sexual systems from tens of thousands of species across plants, vertebrates and other animals. The snapshot of data in Dryad is complemented by a living online database hosted by the consortium (http://purl.org/nescent/treeofsex).

The contribution from Conner *et al.*^8^ presents the detailed results from a long-term artificial selection experiment on floral traits. It includes data that underlie existing publications together with data that have yet to be analyzed and published. A NESCent sabbatical fellowship allowed the corresponding author to compile these years of results, representing a significant fraction of his professional career, into a well-documented whole that can be built upon by others.

The two other contributions in the initial collection, from Zehr *et al.*^9^ and Plooij *et al.*^10^ make available for reuse uniquely valuable observational data from primates. Zehr *et al.*^9^ report the life history data for 3,627 captive individuals from 27 different strepsirrhines (lemurs, lorises and galagos). The data are particularly valuable in combination with the large collection of associated biological samples and the live research colonies at the Duke Lemur Center. Plooij *et al.*^10^ describe the largest dataset of recordings from free-living juvenile chimpanzees, originally collected at Gombe National Park, Tanzania in the early 1970s. The original recordings of 16 animals have been digitized by the Macauley Library and annotated with detailed contextual field notes. These are now available from both the Macaulay Library and in raw form from Dryad. Such irreplaceable datasets are clearly deserving of careful documentation, preservation and stewardship.
